# Are clean energy markets efficient? A multifractal scaling and herding behavior analysis of clean and renewable energy markets before and during the COVID19 pandemic

**DOI:** 10.1016/j.heliyon.2023.e22694

**Published:** 2023-11-22

**Authors:** Bilal Ahmed Memon, Faheem Aslam, Shakhnoza Asadova, Paulo Ferreira

**Affiliations:** a School of Business and Economics, Westminster International University in Tashkent, Uzbekistan; b Department of Management Sciences, COMSATS University Islamabad, Pakistan; c VALORIZA—Research Center for Endogenous Resource Valorization, 7300-555 Portalegre, Portugal; d Department of Economic Sciences and Organizations, Polytechnic Institute of Portalegre, 7300-555 Portalegre, Portugal

**Keywords:** Multifractal detrended fluctuation analysis, Generalized Hurst exponent, COVID19, MLM, Herd behavior, Clean and renewable energy

## Abstract

The literature lacks thorough and adequate evidence of the efficiency and herding behavior of clean and renewable energy markets. Therefore, the key objective of this paper is to explore the multifractality and efficiency of six clean energy markets by applying a robust method of Multifractal detrended fluctuation analysis (MFDFA) on daily data over a lengthy period. In addition, to examine the inner dynamics of clean energy markets around the global pandemic (COVID19), the data are further divided into two sub-periods of before and during COVID19. Our sampled clean energy markets exhibit multifractal behavior with a significant impact on the efficiency and intensified presence of multifractality during the COVID19 period. Overall, TXCT and BSEGRNX were the most efficient clean energy markets, but the ranking of TXCT deteriorated significantly in the sub-periods. The presence of multifractality and herding behavior symmetry intensified during the crisis period, which gives a potential for advancing portfolio management techniques. Moreover, our study provides practical implications and new insights for various market participants for better management and understanding of risks.

## 1 Introduction

In recent times, countries across the globe have been pledging to reduce the reliance on dirty energy (e.g., fossil fuels) and to boost clean energy. The Paris climate agreement of 2015 is one such example, where many countries vowed to replace fossil fuels and invest heavily in sources of clean energy. According to Ref. [1], renewable energy showed the highest growth in 2020, with the majority of countries inclined towards renewables. In addition, the report mentioned a major amount of USD 303.5 billion global new investment in renewable power and fuels. Several studies have reported a tremendous increase in the clean energy markets due to a specialized focus from investors and policy-makers [ [[2], [3], [4]]]. Given the increased attention paid to clean energy markets, it is important for investors and policy-makers to broaden their understanding of clean energy markets’ efficiency and herding behavior.

The analysis of clean energy markets provides numerous implications for the wider energy domain, for example, sustainable development utilizing renewable energy sources, the shift from dirty energy, and advancement of clean energy technologies. In addition, external shocks and crisis periods (e.g., COVID19) present a challenging perspective and influence investor sentiments and market efficiency. While COVID19 has damaged the global economy, the reliance on clean and renewable energy has increased greatly compared to a decline in fossil fuels [5]. Therefore, ranking the efficiency of clean and renewable energy markets during normal and stressful periods is timely and serves as a useful tool for policy-makers and market regulators.

Prior research has documented many approaches to examining the efficiency and stability of clean energy markets [for example [2,[6], [7], [8]]]. However, the question of the informational efficiency of clean energy market remains controversial. The efficient market hypothesis (EMH) implies that investors are unable to exploit the possibilities of abnormal profit out of arbitrage, due to an efficient market where price reveals all the possible information instantly and entirely. Moreover, it is nearly impossible to predict the price due to the lack of exact patterns of asset price. The availability of multifractality represents a pattern (such as a sudden increase or decrease in prices), combined with volatility of prices which presents a likelihood of prediction, thus offering a prospect for some investors to outperform the market. Therefore, the extent of multifractality and market inefficiency, where investors can exploit opportunities, provide several directions for regulators to act immediately and draw up relevant policies to maintain market efficiency, and to overcome the possible alterations in the economy.

In this context, several studies have focused on measuring the extent of multifractality and efficiency of stock markets [ [[9], [10], [11], [12], [13], [14], [15]], bond markets [ [16,17]], foreign currency markets [18,19] commodity markets [ [20,21]], renewable energy [22], and cryptocurrencies [ [[23], [24], [25]]]. Moreover, the highs and lows of the market mechanisms during stress and normal periods reveal hidden features of the markets in exploring their dynamics and herding patterns, where investors behave differently during each market state [26]. Therefore, a comparative analysis and ranking the efficiency of clean energy markets before and during the COVID19 pandemic offers groundbreaking evidence for investors and policy-makers to assess the market correctly. In addition, COVID19 was a worldwide phenomenon with a severe impact on international financial markets, which motivated us to examine the market efficiency and herding behavior before and during the pandemic. That pandemic is considered a high impact once-in-a-generation threat that has challenged the very limits of global financial stability [27].

The multifractal detrended fluctuation analysis (MDFA) method is a robust measure and an extension of the DFA by Ref. [28] to assess the (in)efficiency of the market in a subsequent process. First, the market efficiency of clean energy is measured using various scaling exponents in each of the nonlinear time series. We also discover whether multifractality happened due to long-term temporal correlations or fat-tailed divisions. Another step is to compute the Hurst exponent, which authenticates the availability of multifractality. Finally, the ranking of clean energy indices through the Magnitude of Long Memory (MLM) indexing method illustrates the period-wise dynamics of the market. To sum up, the MFDFA method provides a complete outlook to explore markets that may contain multifractality and provides detailed support to evaluate the efficiencies of various clean energy markets in this study.

The above provides a strong basis on which to conduct this study and rank the efficiency of various clean energy markets before and during COVID19, and overall, to reveal inner dynamics during bullish and bearish market states. Therefore, the present work contributes to literature in many aspects. First, this study examines the multifractality of clean and renewable energy markets thoroughly by focusing on varied multifractal properties through both market states. The examination of multifractal properties before and during the COVID19 pandemic provides enough evidence for market participants to assess the market correctly and take informed decisions, especially during a crisis event. In addition, knowing the price efficiency of clean energy markets assists policymakers in devising and implementing the policies required for a more efficient and buoyant clean energy market combining with the distribution of enhanced resources. Moreover, the study provides valuable insights into the dynamics of clean and renewable energy markets by using subperiods, shedding light on how they were influenced by the unprecedented circumstances of the global pandemic. Second, our study provides a comprehensive and comparative view of clean energy markets. While looking at the variety of clean energy markets, we carefully considered six main indices to measure their efficiency and ranking, which may improve risk management. Third, this paper contributes by applying the robust MFDFA method to clean and renewable energy markets, demonstrating the multifractal scaling properties of these markets and thus extending understanding of market dynamics in this specific sector. Fourth, the analysis in this paper identifies and assesses the presence of herding behavior among market participants in clean and renewable energy markets, particularly during the COVID-19 pandemic. Thus, this contribution highlights the psychological factors influencing market behavior. Finally, this paper introduces application of the MLM technique to rank clean energy markets, which is a rigorous way to assess and compare the performance of various clean energy indices, aiding investors and policymakers in decision-making, and providing insights into the sector's resilience and responsiveness to global crises.

## 2 Literature review

There is little literature analyzing the multifractality and efficiency of clean energy markets comparing a normal and crisis period, and few studies examined the asymmetry and multifractality in numerous financial markets [29]. assessed multifractality among emerging stock markets in the key central and eastern European countries. In addition to finding a varied Hurst coefficient with the q coefficient, their results revealed an influence of the global financial crisis on the overall shape of the multifractal spectrum. Similarly [30], showed that returns of the seven stock indexes belonging to central and eastern European countries have long-range correlations, and thus concluded that these stock markets have not yet matured. In addition [31], detected asymmetric multi-fractal behavior of the US stock market with higher asymmetry being recorded specifically during the financial crisis. Further [32], used the MFDFA method on the Chinese foreign exchange market between July 2005 and April 2009. Their results revealed multifractal characteristics along with both small and large fluctuations due to various dynamic mechanisms [33]. studied multifractal patterns of the return and volatility series of Bitcoin and gold and revealed persistence behavior along with a high extent of Bitcoin multifractality compared to gold [21]. performed a detailed examination using twelve commodity markets over a long timeline covering both major crisis periods of GFC 2007-09 and COVID19. Their results revealed varied multifractality among the entire sample combined with improved efficiency ranking for silver and gold during the crises, thus confirming the evidence of safe-haven hedging instruments. Recently [34], performed a comparative analysis among the foreign exchange rates of five BRICS countries between January 2009 and December 2021. Their results demonstrated considerable differences in efficiency, finding the South African currency to be most efficient and China to be least efficient during the period of study.

In general, the literature focuses mostly on crude oil, given its strategic importance in the energy market. For example [35], examined the random walk hypothesis for the crude oil market using data between 1982 and 2008. The authors documented that inefficiency in WTI crude oil compared to weak-form efficiency in the Brent crude oil market. In addition [36], performed a lagged detrended fluctuation analysis using spot prices of crude oil from 1986 to 2009. Besides showing key deviations from efficiency related to lagged autocorrelation, their results revealed evidence of price reversion to a constantly evolving mean. The results of [37] demonstrated an efficient crude oil market for the entire period, whereas the crisis events led to a decline in the market's efficiency [38]. exhibited the weak-form efficiency and unpredictability of Brent crude oil, while [39] found semi-strong efficiency of the energy market with the absence of true reflection of public information among energy prices.

While assessing the dynamic dependence among crude oil and clean energy stocks [40], used the multivariate GARCH model on the daily data between May 2005 and April 2015. In addition to highlighting the importance of technology stocks in the return and volatility spillover, their results showed that the clean energy index offers a profitable hedging opportunity combined with crude oil futures [41]. adopted a multivariate quantile dependence approach and found oil and electricity prices among the major contributors to the dynamics of clean energy stock returns for the USA and the EU. In addition [42], revealed a significant spillover effect of extreme events among oil and clean energy markets. According to Ref. [43], the return and volatility connectedness is short-lived up to five days among stock prices of U.S clean energy companies and crude oil while establishing a short-lived minor connectedness effect among them in the long run [44]. analyzed the relationship among oil prices and clean energy stock prices, and technology stock prices using weekly data and using Markov-switching vector autoregressive models. Their results found a structural change during the crisis event of 2007, and a positive relationship among oil prices and clean energy prices after structural breaks [45]. found significant time-varying average and symmetric tail dependence among oil returns and various global and sectoral renewable energy indices, providing various implications for risk management and policy assessments [46]. indicated a greater correlation between energy companies and technology stock prices [47]. found major change in the dynamic and static volatility connectedness among crude oil and clean energy firms during the global COVID19 pandemic. Recently [48], employed a quantile VAR approach to examine the degree of spillover among nine indices during various market states. They found clean energy and new energy innovation industry indices to be major drivers of the transmission spillover network during extreme market states. In summary, these studies confirm the importance of analyzing the dynamic effects of market spillover among clean energy, oil, Bitcoin, and financial markets for informed decision-making, risk management, and broader economic and policy implications of these market interactions.

Moreover, some strands of research focus on examining the association between various market indices and prices with the clean energy indices. The initial study was by Ref. [49] using a vector auto-regression model, their results highlighting the impact of oil process changes related to movements in clean energy returns. In addition [50], found unidirectional connectedness from the implied volatility indices to the clean energy stocks. Then again [51], utilized the quantile regression method and showed decreasing dependence of clean energy indices on oil returns [52]. used an Econophysics-based DFA approach to study the behavior of three varied indices including the S&P clean energy index. Their results found greater time serial dependence of the clean energy index along with lower exposure to oil price compared to the NYSE composite index [53]. found a positive link between clean energy development and economic growth [54]. found that an integration of uncertainty indices with global economic conditions can predict clean energy realized volatility. In addition [55], examined the effects of extreme shocks on clean energy stocks, and used an extended model to successfully predict the volatility of clean energy stocks. COVID19 has had a considerable influence globally, hampering the world economy [56]. Economic uncertainty increases substantially in volatile, extreme periods. Therefore, our study offers a rigorous examination of efficiency and herding behavior, covering a wide time span and including a comparative analysis for the period before and during the COVID19 pandemic.

## 3 Data and methodology

We use daily prices of the six major clean energy indices: S&P global clean energy index (S&PGCE), NASDAQ clean Edge green energy index (CELS), S&P/TSX renewable energy and clean technology index (TXCT), S&P BSE GREENEX (BSEGRNX), NASDAQ OMX renewable energy generation (GRNREG), and European renewable energy index (ERIX). The data for these indexes are taken from Bloomberg and Investing.com, for the period until December 2021, as mentioned in Table 1. The sample covered a wide range and included a black swan event of the global COVID19 pandemic. Therefore, the sample period is further divided into two sub-periods of Before-COVID19 and During-COVID19 for a better comparative examination between the normal and extreme periods.

**Table 1:** Description of Clean energy market indices, symbols, and range.

S.no	Symbol	Stock Index	Currency	Beginning Date	Observations
1	GCE	S&P Global Clean Energy Index	USD	22-Oct-13	2146
2	CELS	NASDAQ Clean Edge Green Energy Index	USD	29-Nov-11	2579
3	TXCT	S&P/TSX Renewable Energy and Clean Technology Index	CAD	26-Mar-10	2933
4	BSEGRNX	S&P BSE GREENEX	IND	23-Feb-12	2418
5	GRNREG	NASDAQ OMX Renewable Energy Generation	USD	15-Oct-10	2866
6	ERIX	European Renewable Energy Total Return	EUR	18-Sep-17	1073

[57] mentions that the advancement of renewable energy has significantly aided the stock market. We choose the largest renewable index in Europe (i.e., ERIX), comprising wind, solar, biomass, and water energy generation. In addition, the future prices of the European territory are elected to match ERIX and rebalanced every quarter. The S&P global clean energy index (GCE) comprises a combination of clean or renewable energy equipment and technology companies along with clean energy production companies and provides a platform for liquidity and tradable exposure for around 30 firms. The Nasdaq clean edge green energy index (CELS) is formed of several companies belonging to manufacturing, distributing, and installation of renewable energy technology. The S&P/TSX renewable energy and clean technology index (TXCT) contains a core group of companies responsible for developing green technologies in addition to sustainable infrastructure solutions. The Nasdaq OMX renewable energy generation index (GRNREG) is specific to performance of energy production companies using a variety of sources including solar, wind, geothermal, wave, and fuel cells. Finally, S&P BSE Greenex index (BSEGRNX) measures the performance of 25 key green companies selected from the S&P BSE 100 in terms of size, greenhouse gas (GHG) emission, and liquidity. The empirical analysis is performed by acquiring the natural logarithm of the first difference of two successive daily prices as in equation (1):

(1) $$r_{i}\left(t\right)=\ln\;P_{i}\left(t\right)-\ln\;P_{i}\left(t-\Delta t\right)$$

Where $P_{i}\left(t\right)$ signifies the price series at time t, and $r_{i}\left(t\right)$ indicates the return of the clean and renewable energy index. Usually, market participants demonstrate varied reactions to a good or bad news inflow in the market, leading to possible asymmetric behavior and resultant varied efficiency level for the market [58].

The efficiency of clean energy indices can be examined through the MFDFA [28]. Through this method, the random walk characteristics of the market are determined by generalized Hurst exponents, which helps explain the clustering behavior of the negative and positive tails and evaluates the tendency of the financial time series to regress in relation to the mean. In addition, MFDFA allows better quantification of the data in capturing the fluctuations and allows us to detect the pattern of multifractal behavior [15] which is essential for a thorough investigation during each economic state [59]. In the context of clean energy market indices, this is valuable because the markets are influenced by several factors, and MFDFA can reveal valuable information for both short-term and long-term dynamics. Further, MFDFA can provide quantitative measures of market behavior, including the Hurst exponent and multifractal spectrum [60]. These measures offer insights into the degree of persistence, long-range correlations, and multifractal properties of clean energy market indices, helping analysts and investors to understand market dynamics better. Moreover, risk management is a common focus for market participants involved in the clean energy market, including investors and policymakers. Therefore, the MFDFA method is useful in assisting and evaluating the risks associated with clean energy investments and offers insights into the market's fractal properties, which can be linked to volatility and potential financial risks. The MFDFA method can be obtained using five steps, according to equations 2–11, as summarized below.

Step 1 Construct the corresponding profile of the signal $X_{t}\left(t=1,2,\ldots ,N\right)$ of finite length N by integration as follows in equation (2):

(2) $$Y\left(i\right)=\sum_{t=1}^{i}\left[X\left(t\right)-\left\langle X\right\rangle \right]$$

Where, i=1,2,…,N and $\left\langle X\right\rangle =\frac{1}{N}\sum_{t=1}^{N}X\left(t\right)$ represents the mean value of the entire time series.

Step 2 The series is divided into $N_{s}=\mathrm{int}\left(N/s\right)$ non-overlapping windows of same size s. Moreover, a certain part of the profile remains in most cases as the length N does not need to be a multiple of the considered segment s. To take this part into account, the procedure is repeated commencing from the other end of the profile, and therefore resulting in $2N_{s}$ segments [61].

Step 3 Calculate the local trend $y_{\upsilon }\left(i\right)$ against every window $\upsilon =1,2\text{,}\ldots ,2N_{s}$ through least square fit. Thus, the variance is given as follows in in equations (3), (4):

(3) $$F^{2}\left(S,\upsilon \right)=\frac{1}{S}\;\sum_{i=1}^{S}{\{Y\left[\left(\upsilon -1\right)S+i\right]-y_{\upsilon }\left(i\right)\}}^{2}$$

For each segment $\upsilon =1,2\text{,}\ldots ,N_{s}$ and using

(4) $$F^{2}\left(S,\upsilon \right)=\frac{1}{S}\;\sum_{i=1}^{S}{\{Y\left[N-\left(\upsilon -N_{s}\right)S+i\right]-y_{\upsilon }\left(i\right)\}}^{2}$$

For each segment $\upsilon =N_{s}+1\text{,}\ldots ,2N_{s}$. At this point, $Y_{\upsilon }\left(i\right)$ represents the fitting n-order polynomial in segment v, commonly known as MFDFA-n. The usual choice is for 2n+2≤s≤N/4. For n-order of MFDFA, trends of order n in the profile and n−1 in the primary record are removed.

Step 4 Averaging yields the q th-order fluctuation function given by equation (5):

(5) $$F_{q}\left(S\right)={\left\{\frac{1}{{2N}_{S}}\sum_{\upsilon =1}^{{2N}_{S}}{\left[F^{2}\left(S,\upsilon \right)\right]}^{\frac{q}{2}}\right\}}^{\frac{1}{q}}$$

Here, the q th order can take any real nonzero value, and for q=0, the fluctuation function is given by equation (6):

(6) $$F_{0}\left(S\right)=\exp\left\{\frac{1}{{4N}_{S}}\sum_{\upsilon =1}^{{2N}_{S}}\ln\left[F^{2}\left(S,\upsilon \right)\right]\right\}$$

By using the parameter q, we can distinguish among segments having higher or lower fluctuations. In addition, the estimation of q=2 may retrieve a standard DFA procedure.

Step 5 lastly, the scaling behavior of the fluctuation is attained through evaluating the log-log graphs of $F_{q}\left(S\right)$ against S for the specific q. In case of the availability of long-range power-law correlations in the return series, $F_{q}\left(S\right)$ will increase as a power law concerning S. For large values of S, it is described as equation (7),

(7) $$F_{q}\left(S\right)∼S^{h\left(q\right)}$$

The behavior of clean and renewable energy return series can be evaluated with the Hurst exponent expressed as $h_{q}$. In addition, h(2) is a common Hurst exponent where MF-DFA turns into DFA, but it explains the fractal structure of the given times series. However, if 0.5<H<1, the return series shows persistence, meaning that the present change will have a constant impact on subsequent changes. Subsequently, if 0<H<0.5, the series shows anti-persistent characteristics, indicating strong fluctuations. Moreover, H=0.5 signifies that the index follows an uncorrelated Brownian motion or a random walk behavior, which shows the efficiency of the energy market. In the case of q>0, H(q) signifies higher scale fluctuations, compared to q<0, H(q) which shows lower scale fluctuations. As mentioned by Ref. [62] the Renyi exponent τ(q) can be acquired with the association of h(q) as shown in equation (8):

(8) τ(q)=qh(q)−1

The singularity strength α (Holder exponent) and related singularity spectrum f(α), in addition to legendre transform, is obtained as follows in equations (9), (10):

(9) $$\alpha =\frac{d\tau \left(q\right)}{dq}$$

(10) f(α)=qα(q)−τ(q)

In this paper we calculated the strength of multifractality using the width of multifractal spectrum given by equation:

(11) $$\Delta H=H_{\max}\left(q\right)-H_{\min}\left(q\right)$$

Where a higher value of width ΔH represents a broader singularity spectrum and greater multifractality.

## 4 Empirical analysis and results

### 4.1 Descriptive statistics

Table 2 shows the descriptive statistics of the six clean energy markets used in this study. The mean returns of all six clean energy markets remain close to zero in all the periods, except for BSEGRNX, which shows negative mean returns before COVID19. In addition, the level of risk varies among these markets.

**Table 2:** Descriptive statistics.

	GCE	CELS	TXCT	BSEGRNX	GRNREG	ERIX
Overall						
Mean	0.00035	0.00079	0.00024	0.00045	0.00038	0.00079
Std. Dev.	0.01409	0.01823	0.01132	0.01069	0.01146	0.01680
Skewness	−0.66827	−0.54074	−1.09503	−1.01664	−1.07366	−0.69731
Kurtosis	11.02631	7.11091	22.54492	13.57332	16.55141	5.43573
*2018–**2019 (Before COVID-19)**						
Mean	0.00044	0.00040	0.00003	−0.00003	0.00051	0.00086
Std. Dev.	0.00877	0.01290	0.00670	0.00899	0.00687	0.01165
Skewness	0.02160	−0.29048	−0.25252	0.16972	−0.14478	−0.08506
Kurtosis	0.60510	1.22469	2.18209	1.60530	0.67507	2.36196
**2020–**2021 (During COVID-19)**						
Mean	0.00137	0.00226	0.00059	0.00094	0.00089	0.00095
Std. Dev.	0.02275	0.02952	0.02097	0.01505	0.01735	0.02114
Skewness	−0.74764	−0.66465	−1.04476	−1.75377	−1.41814	−0.70152
Kurtosis	5.50547	3.98368	9.56891	15.70537	15.63667	3.55493

The standard deviation, assessing the volatility of the return series, presents different levels, with the highest volatility for the CELS and ERIX markets of 1.29 and 1.16 % before COVID19. However, volatility increased for all the clean energy markets during COVID19, with the most volatile market being CELS (2.9 %), followed by GCE (2.27 %) in the same period. Similarly [63], used various clean and dirty energy indices and showed higher volatilities among them during the crisis times of the COVID19 pandemic. In addition, the oil price collapsed, and COVID19 substantially increased the extent of risk spillover [64], which is evident considering the specific period of market crisis for the clean energy market. The skewness of all the clean energy markets is not zero, with all the series being skewed towards the left during COVID19 and overall. However, prior to COVID19, GCE and BSEGRNX are positively skewed. Moreover, the kurtosis coefficient is higher than 3 for all markets during the overall and pandemic period, representing their fat tailed behavior. However, a low peak can be observed for the entire sample markets, where the kurtosis value of less than 3 is recorded before the COVID19 period. Consequently, the outcomes regarding the extreme events in the clean energy market support the Fractal Market Hypothesis [65] and are in notable contrast to the opposing view presented by the Efficient Market Hypothesis [66]. Therefore, by applying the MFDFA method, the multifractal properties and scaling behavior of clean energy markets can be assessed, providing insights into the market's complexity and dynamics.

### 4.2 Multifractal detrended fluctuation analysis (MFDFA)

**Fig. 1:**
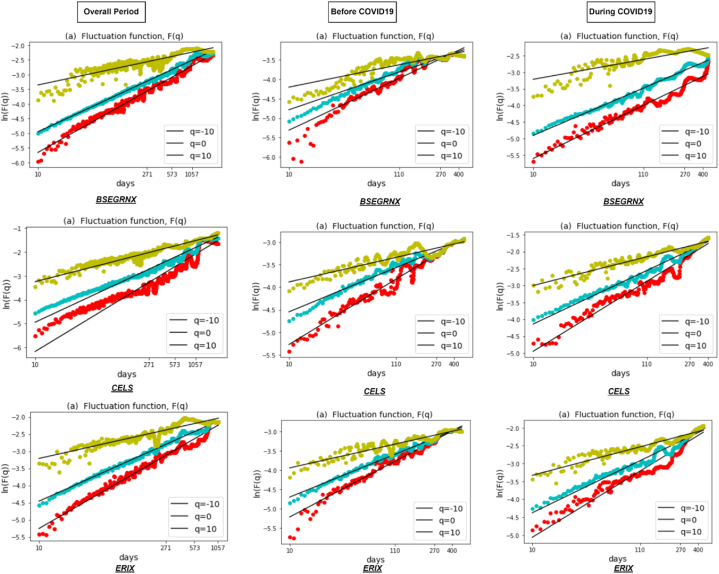

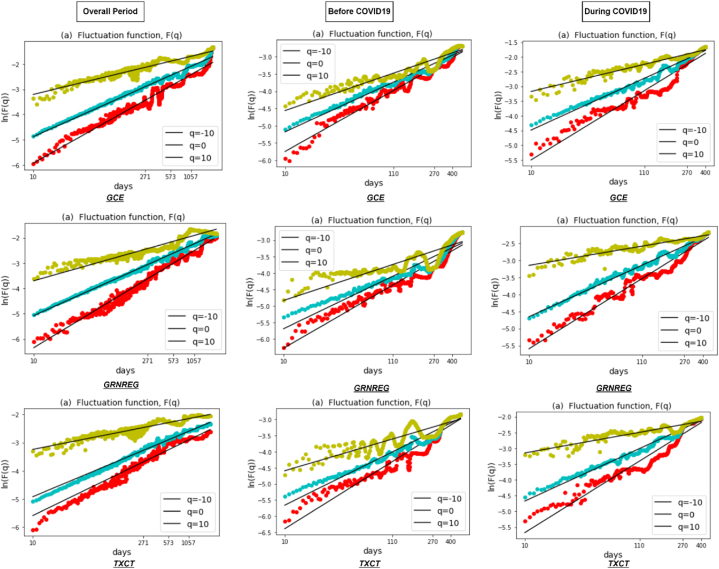
Fluctuation function F(q) for q=−10,q=0,q=10 against each clean energy markets during the overall period and two sub-sample periods.

**Fig. 2:**
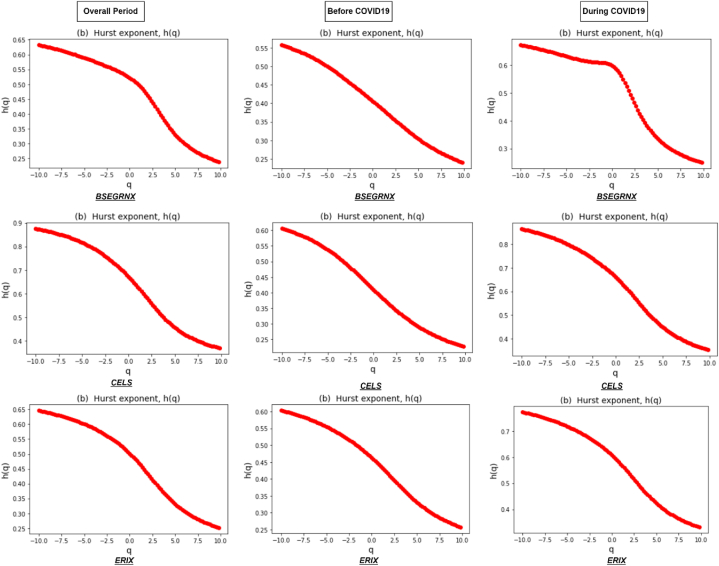

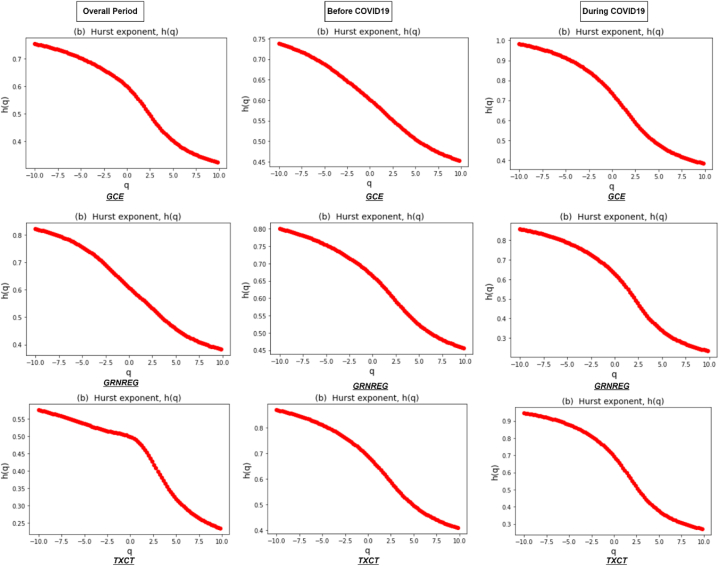
Generalized Hurst exponent depending on q of clean energy markets during the overall period and two sub-sample periods.

Fig. 1 depicts the fluctuation function F(q) of all six clean energy markets during the entire period and sub-sample periods, which are well fitted and show a straight line in the log-log scales. The results represent scaling behavior, with the relationship between the RMS fluctuations and time scales unveiling the multifractal properties of the clean energy market returns. Therefore, the results identify different scaling exponents, indicating the presence of varying degrees of irregularity or fractal patterns in the overall period and during COVID19. Fig. 2 shows the results of the generalized Hurst exponent h(q) over a range of q values from −10to10. This range allows us to capture the impact of both higher and lower variations in the data. The value of h(q) depends on the specific value of q used and provides evidence of multifractality in the returns of the clean energy market. Our results reveal a consistent downward trend of h(q) in all six clean energy markets in all periods, representing the presence of a multifractal pattern.

**Fig. 3:**
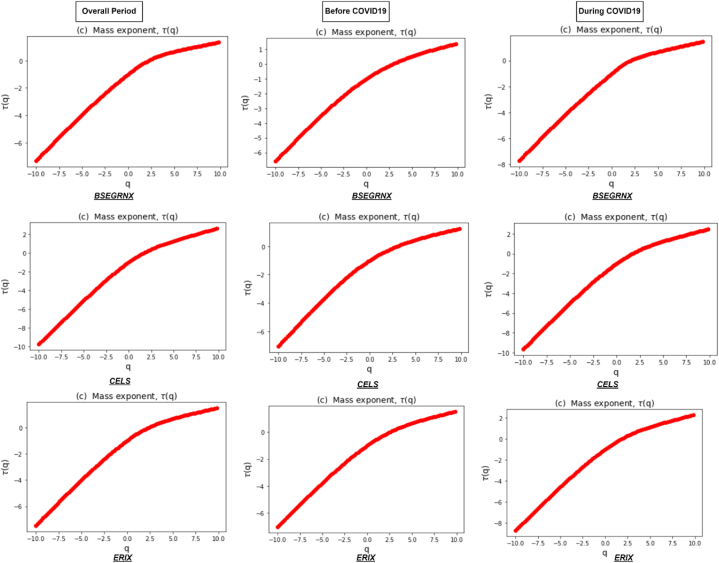

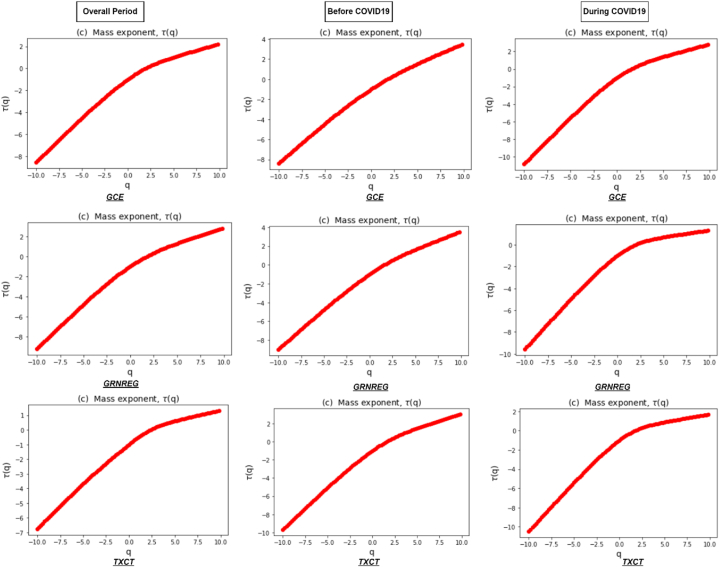
Renyi exponent τ(q) of clean energy markets during the overall period and two sub-sample periods.

**Fig. 4:**
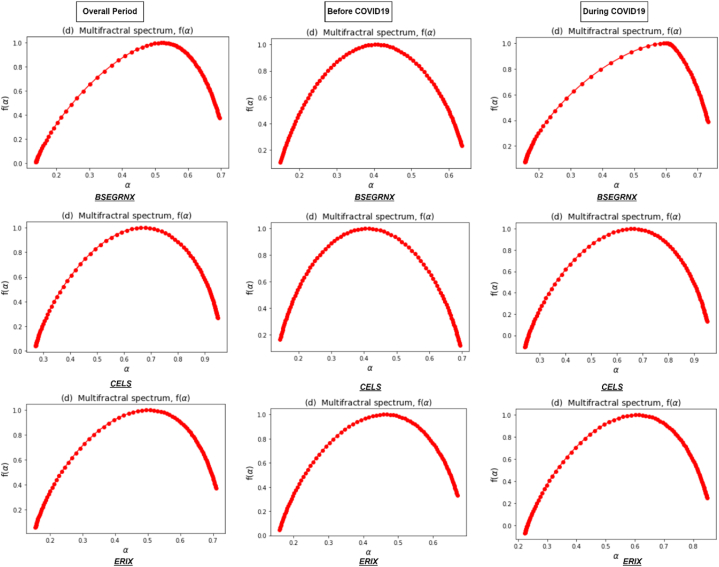

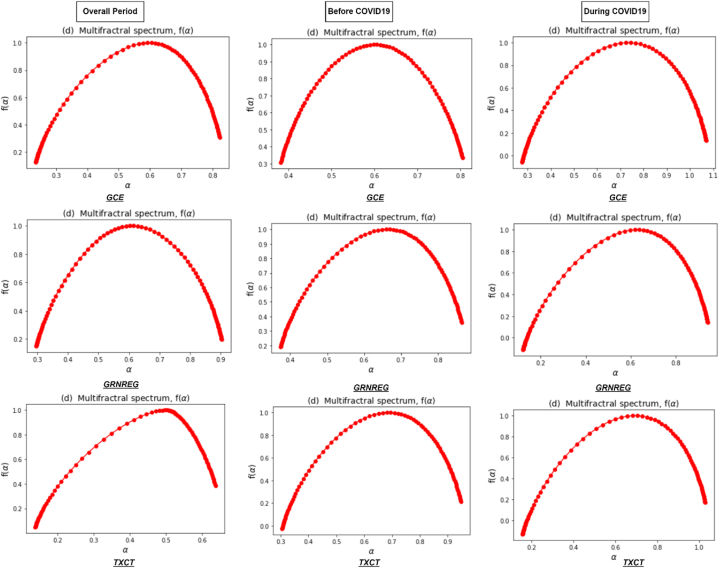
Multifractal spectrum of clean energy markets during the overall period and two sub-sample periods.

Fig. 3 represents the Renyi exponent τ(q), which remains linear for monofractal return series, compared to nonlinear for multifractal series. In addition, the Renyi exponent plays a vital role in recognizing the underlying dynamics of the clean energy markets. Its estimation through the MFDFA method provides a quantitative measure of the multifractal nature of a time series and helps to reveal important properties and patterns within the data. Lastly, Fig. 4 illustrates a multifractal spectrum of clean energy markets during the overall and sub-sample periods. It shows how the fluctuations in the return series are distributed across different scales, indicating the presence of local variations in the data. In addition, it provides a comprehensive view of the system's complexity by revealing the range and intensity of scaling exponents associated with different parts of the signal. A concave shape of multifractal spectrum implies a wide range of scaling exponents, reflecting the existence of multifractality. A flat multifractal spectrum indicates more homogeneous behavior, indicating the absence of multifractality and a more uniform scaling pattern across scales [67].

The clean energy series multifractality results comprising the summary of generalized Hurst exponents over the range of q∈−10to10 during the overall period are presented in Table 3. We immediately observe a declining pattern for all six clean energy markets, representing the availability of multifractality [21]. In addition, we calculated the range Δh that shows the strength of the multifractality, with greater values carrying higher multifractal levels [68]. The widest range is observed for CELS and GRNREG of 0.50793 and 0.44002, respectively, while the narrowest range is noted for TXCT and BSEGRNX of 0.34261 and 0.39547, respectively. Overall, these results show that the most efficient clean market of the entire sample is TXCT, whereas the least efficient clean energy market is CELS. The level of long-range connection is associated with their multifractal characteristics [69]. In addition, the TXCT index is important because it provides a dedicated benchmark for clean energy and technology investments, offers insights into market trends and sustainability efforts, and helps investors make informed decisions in a rapidly evolving economic and environmental landscape [70]. Furthermore, examination of the multifractal properties reveals that TXCT and BSEGRNX demonstrate the least amount of interdependence during the entire period of study.

**Table 3:** Generalized Hurst exponents for six clean energy indices ranging from q∈[−10,10] during the overall period.

Overall						
Order q	GCE	CELS	TXCT	BSEGRNX	GRNREG	ERIX
−10	0.75329	0.87519	0.57500	0.63242	0.82202	0.64632
−8	0.73711	0.85780	0.56115	0.61812	0.80285	0.63181
−6	0.71523	0.83313	0.54438	0.60007	0.77520	0.61268
−4	0.68600	0.79689	0.52627	0.57837	0.73393	0.58700
−2	0.64881	0.74399	0.51169	0.55375	0.67505	0.55189
0	0.59878	0.67166	0.49818	0.52200	0.60854	0.50188
**2**	**0.51827**	**0.58041**	**0.44437**	**0.46191**	**0.54768**	**0.43263**
4	0.43280	0.49048	0.35458	0.36969	0.48482	0.36078
6	0.37729	0.42852	0.29248	0.30150	0.43478	0.30747
8	0.34401	0.39102	0.25579	0.26162	0.40283	0.27274
10	0.32258	0.36726	0.23240	0.23695	0.38200	0.24969
Δ*h*	0.43071	0.50793	0.34261	0.39547	0.44002	0.39663

Table 4 presents Generalized Hurst exponent values for the clean energy markets around the COVID19 pandemic. The values at order q=−10 before COVID19 are 0.695, compared to during COVID19 when there is an increase in the average value of 0.848 for all six clean energy markets. Similarly, the average value of all the sample at order q=10 is 0.339 before COVID19 compared to 0.303 during the COVID19 period. A narrow average range of Δh=0.357 is noted in the pre-COVID19 period, compared to a much wider average range of Δh=0.546 during COVID19. The widest range of Δh is recorded for TXCT before COVID19, and the narrowest width is noted for GCE. However, during COVID19 the range of Δh shows an increase for all markets, and BSEGRNX is the most efficient market as opposed to TXCT which lost its position and became the least efficient of all. During the COVID19 pandemic, global financial markets experienced significant volatility and the BSEGRNX index was not immune to these fluctuations. The BSEGRNX index also experienced a significant drop during this period as investors reacted to the uncertainty surrounding the pandemic and its impact on the global economy. However, governments and central banks implemented various measures to support economies and stabilize financial markets, which helped instill confidence among investors [71]. The BSEGRNX index also exhibited resilience during this phase, reflecting the market's recognition of the long-term potential of environmentally sustainable companies. Some sectors such as renewable energy and clean technology may have experienced a relatively stable or even positive performance due to their long-term growth prospects, while others, such as transportation or hospitality, may have faced more significant challenges.

**Table 4:** Generalized Hurst exponents for six clean energy indices ranging from q∈[−10,10] before and during COVID19.

Before COVID-19 (2018–2019)						
Order q	GCE	CELS	TXCT	BSEGRNX	GRNREG	ERIX
−10	0.73790	0.60572	0.86846	0.55763	0.79997	0.60314
−8	0.72231	0.58504	0.85017	0.53982	0.78510	0.58772
−6	0.70093	0.55643	0.82556	0.51589	0.76536	0.56745
−4	0.67218	0.51691	0.79231	0.48375	0.73955	0.54071
−2	0.63818	0.46600	0.74794	0.44535	0.70707	0.50652
0	0.60168	0.40939	0.68812	0.40566	0.66463	0.46339
**2**	**0.56142**	**0.35509**	**0.60918**	**0.36382**	**0.60766**	**0.41010**
4	0.52220	0.30868	0.52837	0.32254	0.54797	0.35495
6	0.49083	0.27261	0.46994	0.28709	0.50358	0.31021
8	0.46803	0.24592	0.43225	0.25938	0.47440	0.27804
10	0.45154	0.22623	0.40721	0.23839	0.45476	0.25518
Δ*h*	0.28636	0.37949	0.46125	0.31924	0.34521	0.34796
**During COVID-19 (2020**–**2021)**						
−10	0.98244	0.86351	0.94563	0.67150	0.85621	0.77240
−8	0.96183	0.84309	0.92596	0.65758	0.83586	0.75503
−6	0.93236	0.81501	0.89765	0.64048	0.80713	0.73204
−4	0.88923	0.77647	0.85541	0.62212	0.76604	0.70173
−2	0.82639	0.72590	0.79182	0.61026	0.70872	0.66185
0	0.73486	0.66031	0.69427	0.59809	0.62842	0.60775
**2**	**0.61686**	**0.57406**	**0.55258**	**0.49401**	**0.50669**	**0.53480**
4	0.51420	0.48523	0.42117	0.37299	0.38151	0.45625
6	0.44855	0.42081	0.34243	0.30760	0.30451	0.39589
8	0.40850	0.37953	0.29738	0.27126	0.26034	0.35628
10	0.38256	0.35238	0.26935	0.24844	0.23281	0.33013
Δ*h*	0.59989	0.51113	0.67628	0.42305	0.62340	0.44228

As mentioned by Ref. [72], the Hurst exponent is proposed as a reliable metric for capturing herding behavior within a time series. Subsequently, various studies have used this useful measure to examine the herding behavior of various markets [ [21,25]]. The degree of persistence exhibited by return series is identified as a significant factor influencing multifractality. Looking at the ranges of the classical Hurst exponent (q=2), we observe that before COVID19, three markets of ERIX, BSEGRNX, and CELS show anti-persistent behavior (H<0.5), representing a tendency to revert to long-term mean or constantly convert themselves. However, the remaining three markets of TXCT, GRNREG, and GCE displayed persistent behavior (H>0.5), indicating positive autocorrelation and providing evidence of herding behavior. It further suggests that traders increased their herding behavior, indicating the presence of positive feedback-based herding behavior among these three clean energy markets. However, during COVID19, almost all the markets show persistent behavior (positive autocorrelation) with traces of herding, except for BSEGRNX. In this scenario, a positive (negative) return observed within a specific time period is likely to be succeeded by a negative (positive) return on the subsequent day. Moreover, it is essential to mention that while markets can exhibit persistent behavior during crises, they can also be highly unpredictable at such times [ [73,74]]. However, the results of our study confirm the existence of herding, which is not unusual and in line with the results of previous studies that found significant herding behavior specifically during extreme events [ [[75], [76], [77], [78]]].

### 4.3 Ranking the clean energy markets

To establish the findings’ ranking and robustness, the effectiveness of clean energy markets is additionally assessed using the MLM measure, which ranks them based on their efficiency. Greater MLM values indicate a higher degree of market inefficiency. Table 5 summarizes the results of average Hurst and MLM during the overall and two sub-sample periods for all the clean energy markets. The results demonstrate that TXCT was the most efficient market during the overall period, but it fell in the ranking specifically in the sub-sample periods to last place, representing the least efficient clean energy market during these periods. GCE was the most efficient clean energy market just before COVID19 and continued in the middle during COVID19 and the overall period. The COVID19 pandemic has presented a significant growth potential for sustainable finance [79]. In addition, clean energy indices typically evaluate various parameters such as renewable energy capacity, energy efficiency, policy frameworks, and investment in clean energy [80]. Through efficiency ranking, policy-makers, businesses, and other stakeholders can identify specific areas that require attention and improvement.

**Table 5:** Results of clean energy markets’ efficiency.

Market	Overall			Before COVID-19			COVID-19		
	Average Hurst	MLM	Ranking	Average Hurst	MLM	Ranking	Average Hurst	MLM	Ranking
GCE	0.55765	0.21535	4	0.59702	0.14318	1	0.69980	0.29994	4
CELS	0.63967	0.25397	6	0.41346	0.18974	5	0.62694	0.25556	3
TXCT	0.43603	0.17130	1	0.65632	0.23063	6	0.63579	0.33814	6
BSEGRNX	0.46695	0.19774	2	0.40176	0.15962	2	0.49948	0.21153	1
GRNREG	0.60634	0.22001	5	0.64091	0.17261	3	0.57166	0.31170	5
ERIX	0.46863	0.19832	3	0.44340	0.17398	4	0.57310	0.22114	2

## 5 Conclusion and policy implications

The COVID19 pandemic is considered one of the greatest challenges to the global economy since World War II. It has caused massive disruptions and led to unprecedented levels of deficit spending to revive economic activity due to widespread infections and subsequent lockdown measures. In addition, the pandemic has had prolonged impacts on the global financial markets. Thus, pessimistic investors tried to imitate the behavior of a rational, informed investor, resulting in a psychological state of behavioral biases including herding. Despite growing public awareness and interest in renewable energy, clean energy companies have relatively low market valuations compared to traditional energy industries. This is partly due to the perception that the clean energy sector is still emerging and untested, creating uncertainty and perceived risks for investors.

This study provides a thorough examination of the efficiency and herding behavior of six major clean and renewable energy markets using an overall and two sub-sample periods around the global COVID19 pandemic. We used the MFDFA methodology and the Generalized Hurst Exponent along with the robust measure of magnitude of Long Memory to measure the varied extent of the multifractality, long memory, and efficiency ranking of these markets. The MFDFA method is specifically designed to detect and quantify multifractal scaling properties in time series data [81]. Therefore, it reveals the presence of multifractal behavior, which signifies complex and heterogeneous dynamics, making it suitable for capturing the intricate characteristics often found in financial and economic time series. In addition, the method is robust to nonlinearity and covers nonlinear patterns and multifractal scaling, which is common in the time series of clean and renewable energy markets. Our results suggest that clean energy markets are not a random process as proposed by EMH, but a process influenced by both higher and lower fluctuations in particular periods. Moreover, we observed a significant presence of multifractal behavior in the clean energy markets during COVID19, when the average range of Δh increased substantially compared to the previous period. An interesting finding was that TXCT was the most efficient market during the overall period, but the least inefficient during the sub-sample periods. CELS and GRNREG were the least efficient markets during the overall period of study. However, CELS efficiency improved during COVID19, while BSEGRNX appeared to be a more efficient clean energy market during the entire period and both sub-periods.

Our study confirms substantial herding behavior during the COVID19 pandemic, confirming the availability of complex patterns that contribute to market inefficiency. The tendency to follow the crowd significantly influences the formation of asset bubbles in clean energy markets, potentially leading to destabilizing consequences. However, the literature review suggests that this behavior may be motivated by rational decisions made by speculators with short-term perspectives, arbitrageurs, or noise traders. This can impact on the effectiveness of information processing and give rise to irrational bubbles. In such scenarios, speculators may prioritize a single information source instead of considering multiple sources, which could ultimately result in greater returns.

Given that the level of multifractality can offer insights into the relative level of advancement among clean energy markets, it is crucial for authorities and regulators to enhance market efficiency. This can be achieved by establishing an institutional framework that promotes long-term, sustainable growth in these specific markets. In addition, the lack of efficiency in the short and sometimes long-term dynamics of the clean energy market offers various advantages to different players within this market. When the prices of constituent companies in these clean energy indices fail to fully reflect all available market information, market participants have the opportunity to incorporate undisclosed information in their investment or management strategies. As a result, the inefficiency of the clean energy market in the short term presents diversification prospects and potential gains for both individual and institutional investors. Further, our results show herding behavior in clean and renewable energy markets, especially during the COVID-19 pandemic, which suggests the need for enhanced regulatory oversight. Policymakers may consider implementing measures to monitor and mitigate the impact of herding behavior to maintain market stability and prevent excessive price volatility. Regulatory bodies can use insights from our study and design investor education programs to minimize the risk. The multifractal scaling analysis can inform policymakers about the inherent complexity and risk associated with clean energy markets. This information can be used to develop risk management strategies, such as creating contingency plans and stress testing mechanisms, to ensure the resilience of clean energy investments in times of crisis. Moreover, asset managers, wealth managers, and clean energy funds, for instance, can employ arbitrage or speculative strategies. Likewise, individual investors and collectors can focus on exploiting the inefficient allocation of specific resources to optimize their portfolios. Insurers and brokers can leverage the market inefficiency in the clean and renewable energy industry to expand their market share. However, during times of crisis such as COVID19, these investors seek reliable and comparable non-financial information to make informed decisions. Therefore, there is a crucial need for robust, standardized information, ultimately leading to greater market transparency. Achieving this enhanced transparency may involve investment in quality through promoting sound cultural practices and reinforcing controls. The ranking of clean and renewable energy markets can lead policymakers to incentivize diversification of clean energy investments across different markets, thereby reducing the concentration of risk in a particular sector or region, as well as providing targeted incentives and support to foster growth in these markets.

While our paper makes valuable contributions to the analysis of clean and renewable energy markets, it is important to recognize the limitations. Firstly, it focuses primarily on identifying multifractal scaling properties, herding behavior, and market rankings. However, it does not explore causality relationships between these factors or investigate the impact of specific policy interventions on market behavior, which could provide a more comprehensive understanding of market dynamics. In addition, we have identified traces of herding in the analysis, but our paper may not provide a comprehensive explanation of the underlying causes and motivations driving herding behavior among market participants during the pandemic. Therefore, additional qualitative research is needed to go deeper into these aspects. Moreover, to comprehend the root causes of multifractality in the clean energy markets, it is recommended that future studies utilize diverse multifractal methodologies. Applying the dynamic rolling window approach over a long period of time is something else that will offer detailed insights into market efficiency.

## Funding

Paulo Ferreira acknowledges the financial support of Fundação para a Ciência e a Tecnologia (grant UIDB/05064/2020).

## Data availability statement

Data will be available on request.

## CRediT authorship contribution statement

**Bilal Ahmed Memon:** Writing – review & editing, Writing – original draft, Visualization, Methodology, Investigation, Formal analysis, Data curation, Conceptualization. **Faheem Aslam:** Writing – review & editing, Visualization, Formal analysis. **Shakhnoza Asadova:** Writing – review & editing, Writing – original draft, Validation. **Paulo Ferreira:** Writing – review & editing, Supervision, Methodology.

## Declaration of competing interest

The authors declare the following financial interests/personal relationships which may be considered as potential competing interests:Paulo Ferreira reports article publishing charges was provided by Fundação para a Ciência e a Tecnologia.
